# Management of cardiogenic shock: a narrative review

**DOI:** 10.1186/s13613-024-01260-y

**Published:** 2024-03-30

**Authors:** Driss Laghlam, Sarah Benghanem, Sofia Ortuno, Nadia Bouabdallaoui, Stephane Manzo-Silberman, Olfa Hamzaoui, Nadia Aissaoui

**Affiliations:** 1 Research & Innovation Department, RIGHAPH, Service de Réanimation polyvalente, CMC Ambroise Paré-Hartmann, 48 Ter boulevard Victor Hugo, 92200 Neuilly-sur-Seine, France; 2 Service de médecine intensive-réanimation, Hôpital Cochin, Assistance Publique-Hôpitaux de Paris (AP-HP), Centre & Université Paris Cité, Paris, France; 3 Université Paris Cité, Paris, France; 4 Service Médecine intensive-réanimation, Hopital Européen Georges Pompidou, Paris, France; 5 Université Sorbonne, Paris, France; 6 Institut de cardiologie de Montreal, Université de Montreal, Montreal, Canada; 7 Sorbonne University, Institute of Cardiology– Hôpital Pitié-Salpêtrière (AP-HP), ACTION Study Group, Paris, France; 8 Service de médecine intensive-réanimation polyvalente, Hôpital Robert Debré, CHU de Reims, Reims, France; 9 Unité HERVI “Hémostase et Remodelage Vasculaire Post-Ischémie” - EA 3801, Reims, France; 10 AfterROSC, Paris, France

**Keywords:** Cardiogenic shock, Shock, Myocardial infarction, Hemodynamic monitoring, Mechanical ventilation, ECMO

## Abstract

Cardiogenic shock (CS) is characterized by low cardiac output and sustained tissue hypoperfusion that may result in end-organ dysfunction and death. CS is associated with high short-term mortality, and its management remains challenging despite recent advances in therapeutic options. Timely diagnosis and multidisciplinary team-based management have demonstrated favourable effects on outcomes. We aimed to review evidence-based practices for managing patients with ischemic and non-ischemic CS, detailing the multi-organ supports needed in this critically ill patient population.

## Introduction

Cardiogenic shock (CS) is a life-threatening syndrome defined by peripheral hypoperfusion and organ dysfunction due to primary cardiac dysfunction. It has several underlying aetiologies, the most common being acute myocardial infarction (AMI). Other less common causes include de novo subtypes of CS (fulminant myocarditis, right ventricular [RV] failure, Takotsubo syndrome, post-partum cardiomyopathy, end-stage valvular heart disease) and acute decompensation of other cardiomyopathies [1].

Despite the many advances in cardiovascular care over the last 20 years, survival of CS patients has not changed substantially and remains around 50% at 30 days following diagnosis [2].

In this review, we aimed to address several aspects of CS, highlighting the importance of understanding CS’s pathophysiology and phenotypes to improve the management of these critically ill patients admitted to the intensive care units (ICU).

### Definitions and classifications

**Fig. 1:**
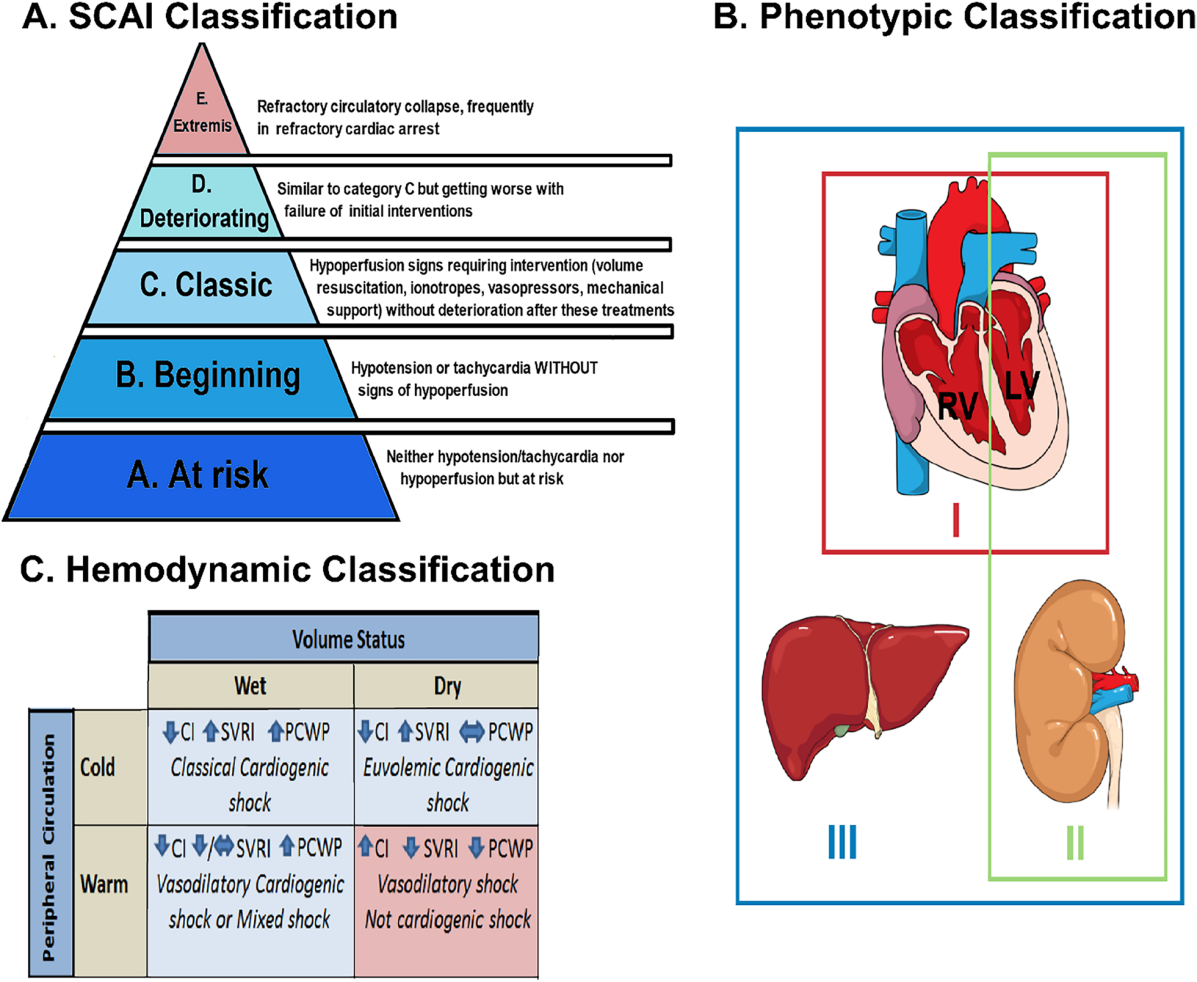
Classifications of cardiogenic shock. **A** SCAI classification. **B** Phenotypic classification: Three different phenotypes are proposed (I-Not congested, II-cardiorenal, and III-cardiometabolic). **C** Hemodynamic classification: CI, cardiac index; PCWP, pulmonary capillary wedge pressure; and SVRI, systemic vascular resistance index

CS is defined as clinical and biological evidence of tissue hypoperfusion secondary to cardiac dysfunction [3]. Although the clinical definition of CS varies, it usually includes hypotension (systolic blood pressure ≤ 90 mmHg) despite adequate filling pressures, and signs of organ hypoperfusion. Most of definitions and classifications have centered on AMI-CS. However, the use of "one size fits all" definitions does not account for the CS hemodynamic phenotypes which can vary from those with myocardial dysfunction due to ischemia requiring minimal vasopressor support to CS with ongoing cardiac arrest. Accordingly, the relatively new classification of the Society for Cardiovascular Angiography and Interventions (SCAI) describes five evolutive stages of CS, from A (preshock, i.e., a patient at risk but with no obvious signs of hypoperfusion) to E (extremis, i.e., refractory circulatory collapse) attempting to better describe the different levels of CS severity Fig. 1a [3]. This classification has been validated in a large cohort of unselected cardiac ICU patients, showing a strong association between SCAI shock stages and mortality, even after adjustment for known predictors of mortality [4]. A second classification focusing on hemodynamic parameters classifies patient into four separates states according to their status volume and their peripheral circulation Fig. 1c [5]. Using this hemodynamic classification and by better characterizing each patient’s “tissue response” to decreased perfusion and the subsequent evolution of specific clinical disease pathways, some authors rationalized that earlier and more specific interventions could be employed to improve CS survival rates [6]. Another classification based on three different phenotypes has been proposed (I-Not congested, II-cardiorenal, and III-cardiometabolic), Fig. 1b [7]. In detail, the non-congested phenotype (I) exhibits lower heart rate, normal filling pressures (right atrial and pulmonary capillary wedge pressures), and a higher blood pressure relative to the other phenotypes, representing a relatively stable profile of a non-congested patient with CS. On the other hand, patients in the cardiorenal shock (II) group are older, with multiple comorbidities. They exhibit a lower heart rate, elevated pulmonary arterial and pulmonary capillary wedge pressures, as well as lower glomerular filtration rate, suggesting renal involvement from shock. Finally, the patients in the cardiometabolic shock (III) group have elevated lactate, alanine aminotransferase, serum creatinine, blood urea nitrogen levels, heart rate, and right atrial pressure, along with low blood pressure, cardiac power output, and index. This suggests multiorgan involvement, featured by increased serum creatinine, blood urea nitrogen levels, transaminases and lactic acidosis in a patient with CS. This classification highlights that CS begins with hemodynamic compromise that triggers impairment of renal and liver function progressively leading to a self-perpetuating “cardiometabolic” shock phenotype causing a further downward spiral.

### Pathophysiology

CS involves several pathophysiologic mechanisms that may have consequences for clinical management. Thus, one has to differentiate de novo or acute cardiac conditions leading to CS and CS related to acute-on- chronic heart failure [8]. De novo CS are mainly due to acute MI (AMI), unstable angina, postcardiotomy syndrome, valvular heart disease, myocardial disease (such as myocarditis), stress-induced cardiomyopathy, sepsis-induced cardiomyopathy, pericardial tamponade, congenital lesions, or/and mechanical injury to the heart. Many triggers lead to acute decompensation of chronic cardiac failure and lead to CS.

Cardiogenic shock arises from an imbalance between input and demand, causing systemic hypoperfusion and organ dysfunction [5, 9–11]. This leads to microcirculatory changes, inflammatory activation, anaerobic metabolism, and oxidative stress, resulting in altered oxygen delivery and microvascular dysfunction that contribute to end-organ dysfunction [12–14].

Distinguishing between acute CS and acute-on-chronic CS is essential because mortality is higher in acute CS [15–17]. It also seems interesting to assess the different haemodynamic profiles in both entities, as this may help to tailor therapies [6, 15].

In acute CS, there is a sudden reduction in ventricular contractility, leading to decreases in stroke volume, CO and blood pressure and increases in pulmonary capillary wedge pressure and central venous pressure. Consequently to systemic hypoperfusion, organ compensatory mechanisms are triggered to restore CO and peripheral perfusion. Among the compensatory homeostatic responses to a drop in CO, the most well-recognized are activation of the sympathetic nervous system and the renin–angiotensin–aldosterone system, secondary to low mean blood pressure and decrease in renal blood flow, respectively. These mechanisms lead to fluid reabsorption and systemic vasoconstriction, increasing volume overload and ventricular afterload and worsening myocardial contractility [5, 11].

Acute-on-chronic heart failure evolves from an initial hemodynamic disturbance into a multisystem disorder. Arterial vasoconstriction secondary to activation of the baroceptors and chemoreceptors increases vascular resistance to peripheral organs and redistributes blood away from the splanchnic circulation. Moreover, venoconstriction increases stressed blood volume resulting in significant increases in central venous pressures in the presence of right ventricular (RV) failure and a variable response in pulmonary venous pressures depending on LV function. Congestion in the setting of CS is also associated with organ dysfunction and poor prognosis; and may be challenging to manage [18]. Indeed, progressive elevations in central venous pressures lead to visceral venous congestion and end-perfusion organ (e.g., kidney and liver) dysfunction. Chronic heart failure progresses to CS when impaired ventricular contractility is severe enough to cause a critical reduction in cardiac output (CO). This reduction of CO may be due to an increase in demand because of a triggering factor or to the natural progression of the disease with a progressive reduction in cardiac output. End organ hypoperfusion results in acute-on-chronic hepatic and renal insults, lactic acidemia, decreased coronary perfusion pressure, and further activation of mechanoreceptors and chemoreceptors, all of which cause a vicious circle of worsening cardiac function [8].

### Epidemiology

Most epidemiological data on shock in critically ill patients focus on severe sepsis and septic shock, which are thought to be the leading causes of mortality in these patients. Although less frequent, cardiogenic shock (CS) remains a genuine clinical challenge with similar or even higher mortality rates [19–21]. Overall, CS patients seem to represent 7–10% of patients admitted to ICU [20]. However, the prevalence of CS is variable according to the type ICU in which patients are being treated and is higher in cardiac ICUS compared to general ICUs.

Data regarding CS epidemiology are predominantly derived from large registries of patients with CS secondary to AMI (AMI-CS). In three nationwide French registries from 2005 to the end of 2015 assessing 9951 patients with AMI, the prevalence of CS has markedly decreased, both for primary (those with CS on admission) and secondary CS (those who developed CS subsequently during hospitalization), so that CS has become an infrequent complication of AMI (2.8%). However, 1-year mortality in patients who developed CS remained exceptionally high (58%) [1].

There are limited datasets that have incorporated CS across all its etiologies; therefore, comparison of outcomes between AMI and non-AMI-CS is still challenging. Data from a French registry suggest that patients with non-AMI-CS may have comparatively better survival rates (36% vs. 31%) [20]. Regarding the effect of cardiac comorbidity, patients with acute cardiac failure tend to have more severe shock presentations, higher lactate levels, and higher Sequential Organ Failure Assessment (SOFA) scores, even if they are less comorbid and have fewer cardio-vascular risk factors than those with CS due to acute-on-chronic heart failure [16, 22]. Consequently, in-hospital mortality appears to be higher in de novo cardiac dysfunction compared to acute-on-chronic heart failure-CS [16, 17]. Intensivists should thus pay attention on such acute cardiac conditions, as it seems to be a strong marker of mortality.

### Management of CS

#### Assessment and diagnosis

**Fig. 2:**
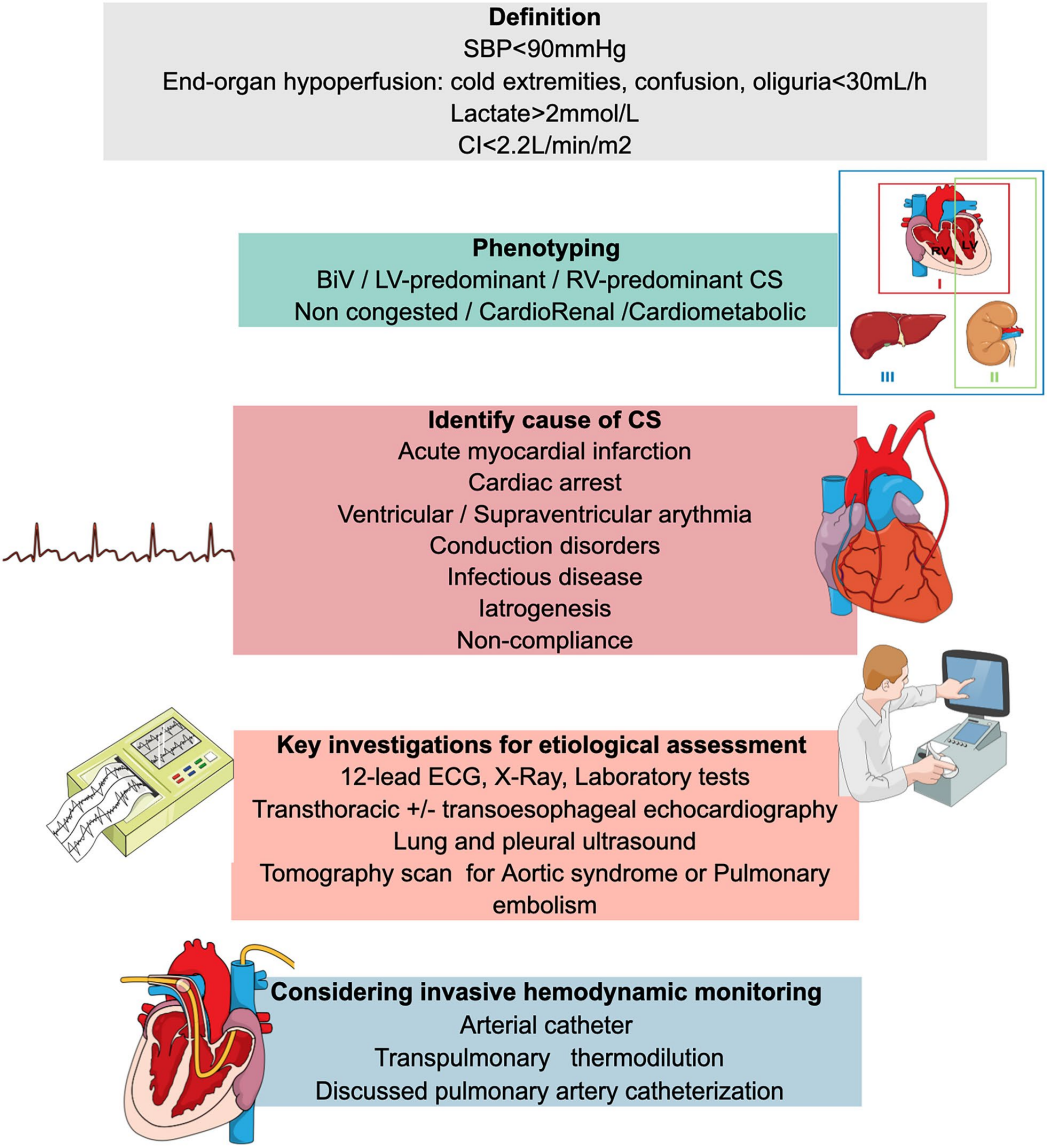
Assessment and diagnosis algorithm for CS early management. *Abbreviations*: BiV, biventricular dysfunction; CI, cardiac index; CS, cardiogenic shock; ECG, electrocardiogram; LV, left ventricular; RV, right ventricular; SBP, systolic blood pressure

The approach to manage CS patients should be multimodal. It includes assessment of severity and early determination of the etiology with clinical examination and non-invasive exploration (clinical ECG, biological and echocardiographic tests). Transthoracic echocardiography (TTE) is the most useful diagnostic modality in this setting, due to its ability to provide comprehensive information about cardiac structure and function, promptly, safely, and at the patient’s bedside. It allows to assess biventricular function, valvular pathology, pericardial effusion, left and right ventricular filling pressure and hemodynamic assessment. Moreover, early comprehensive TTE can provide important prognostic insights in CS patients [23–26]. The invasive hemodynamic assessment may be required to assess response to treatment and for continuous monitoring of cardiovascular function, especially when there is insufficient clinical improvement to initial treatment maneuvers (See Fig. 2). Management of CS requires close collaboration between cardiologists and intensivists, with multidisciplinary discussion and early admission to intensive care.

#### Monitoring

Current guidelines emphasize the initiation of early (within the first few hours) basic monitoring that should be supplemented by advanced monitoring in more complicated cases and in refractory shock [27, 28]. Indeed, during the first hours of CS management, evaluation of organ dysfunction by physical examination (looking for cool extremities, mottling [29], oliguria < 30 mL/h, abdominal pain and signs of encephalopathy), biological tests (including lactate) and echocardiography, as well as invasive arterial pressure measurement allow the early recognition, better classification and precise phenotyping of CS providing robust mortality risk stratification in this population [4, 30, 31].

Lactate level (pathologic threshold > 2 mmol/L) monitoring should be measured on admission and repeated every 1–4 h based on clinical conditions in the early stage of CS management [5, 34, 35]. Elevated lactate level is associated with an increased risk of mortality [36–38]. Conversely, complete lactate clearance is an independent predictor of in-hospital survival as early as 8 h after enrollment in a sub-analysis of DOREMI-trial [39].

TTE is one of the most useful basic monitoring modality in CS patients, due to its accuracy to determine CO and CI with good correlation with pulmonary artery catheters (PAC) data [32, 33]. It also allows to determine left and right ventricular filling pressure (even if several limits exist), pulmonary artery systolic pressure and systemic vascular resistance [33]. On the other hand, trans-oesophageal echocardiography is not the first line of monitoring in these unstable patients. It should be performed under optimal safety conditions, under sedation and by an expert of the technique. Of note, among advanced monitoring modalities, transpulmonary thermodilution technique may be used to directly estimate LV systolic function when TTE is not immediately available [27].

In addition to this standard/basic monitoring, parameters derived from central venous catheter (in the superior vena cava territory) may be also helpful in severe patients: the central venous oxygen saturation (ScvO2), which reflects balance between supply and consumption, and the veno-arterial difference in CO_2_ tension [“ΔPCO_2_” or “PCO_2_ gap”, elevated > 6 mmHg)] a surrogate of the cardiac output because it reflects the balance between CO_2_ production and CO_2_ delivery to the lungs [30, 40]. No specific threshold is reported in current guidelines but goal is increasing of ScvO2 values with serial evaluation (every 4 h) after treatment initiation [5, 31].

Besides, in the most severe patients, PAC could be discussed. It is important to note that the insertion and management of PAC must be carried out by experts, which reinforces the need to transfer patients to an expert CS centre. PAC directly measure pulmonary and cardiac pressures as well as central oxygen saturation (SVO2) and are used to calculate an array of hemodynamic parameters including: cardiac output (CO, L/min) and cardiac index (CI, L/min/m^2^), cardiac power output (Watts, calculated as mean arterial pressure × cardiac output/451) [41], pulmonary capillary wedge pressure (PCWP, elevated > 18 mmHg), right atrial pressure (RAP, elevated > 12 mmHg), mean pulmonary arterial pressure (mmHg and pulmonary artery pulsatility index (PAPi) (ratio of the pulmonary artery pulse pressure to right atrial pressure, predictor of right ventricular failure) [42]. Based on these parameters, physicians can have complete hemodynamic evaluation and categorize CS patients into RV and/or LV dysfunction, right-sided (elevated RAP) congestion, left-sided (elevated PCWP) congestion, bi-sided (both RAP and PCWP elevated) congestion, or euvolemic status (both RAP and PCWP below cutoff values) [15, 43, 44]. An increasing number of studies report the use of PAC and its association with CS prognosis improvement [43, 45, 46]. In this regard, recent guidelines recommend invasive hemodynamic assessment for continuous hemodynamic monitoring in the acute management of patients receiving therapy with MCS, to guide its withdrawal and to supervise the pharmacologic support in patients with myocardial recovery from CS [5, 47]. Finally, in patients without recovery of myocardial and end-organ function, advanced/invasive hemodynamic monitoring is useful to assess candidacy for and/or transition to advanced heart failure therapies, including durable mechanical circulatory support and heart transplantation [5, 47].

### Management

**Fig. 3:**
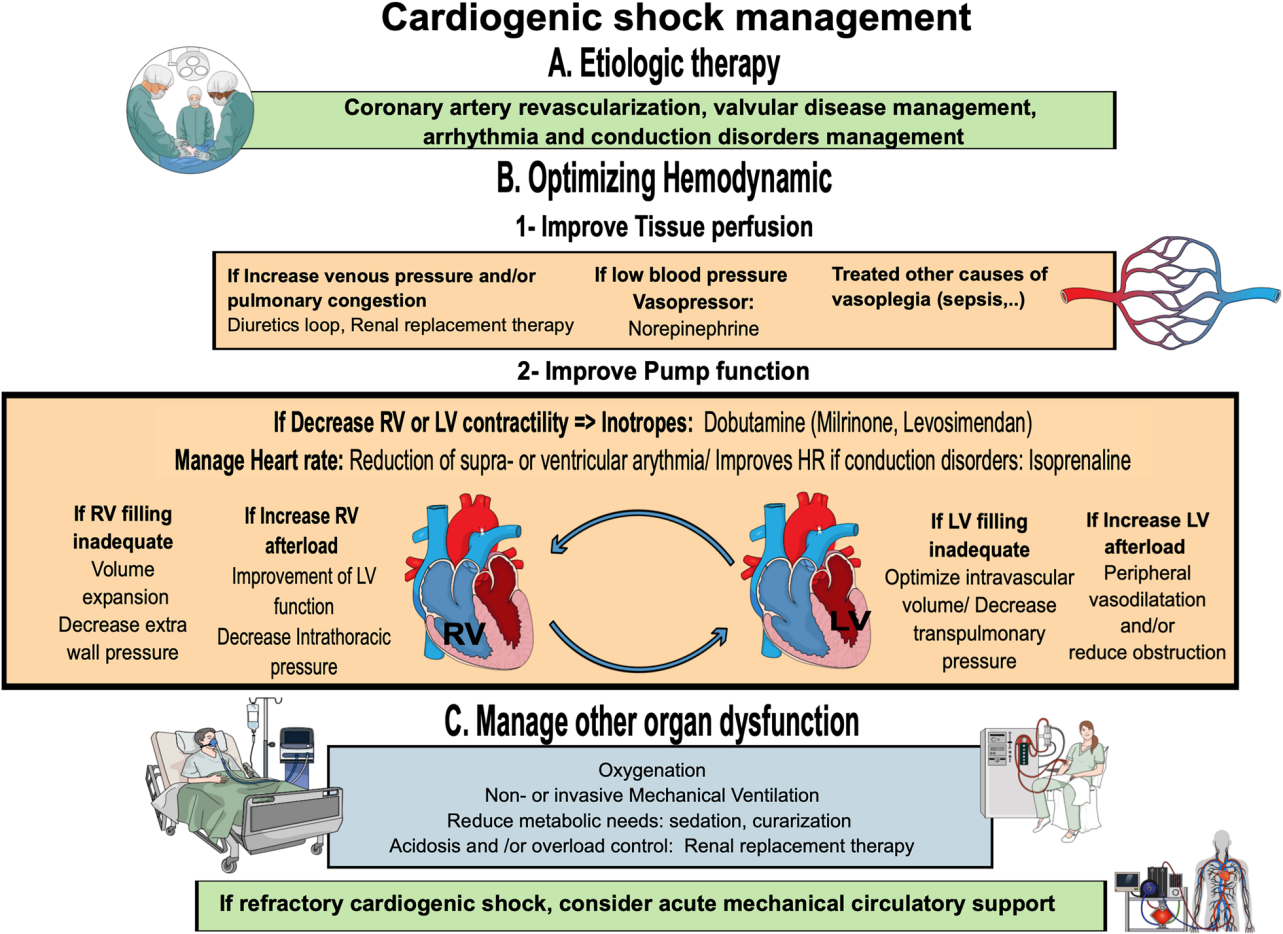
Cardiogenic shock management. *Abbreviations*: AV ECMO, arteriovenous extracorporeal membrane oxygenation; HR, heart rate; LV, left ventricular; RV, right ventricular

Figure 3 summarises the management of cardiogenic shock.

#### Coronary artery revascularization

The cornerstone of treatment that improved CS prognosis in AMI patients is emergent coronary revascularization (or Percutaneous Coronary Intervention PCI) in patients with coronary artery disease [48]. The SHOCK trial highlighted that early revascularization in AMI-CS significantly decreased 6 months (50 vs. 63%, *p* = 0.027) and long-term mortality compared with intensive medical treatment [49]. The benefit of an initial revascularization strategy was confirmed in a nationwide cohort study including 60,833 patients, showing a significant reduction in mortality compared with a conservative strategy. This improvement appears in all patient subgroups, including the elderly [50]. These studies confirmed the need for an initial invasive management approach in case of AMI-CS. Although its implementation reduces the incidence of CS in myocardial infarction [51], fibrinolysis is ineffective once shock has set in, due to reduced penetration into thrombus as there is a decrease in blood pressure. In this regard, fibrinolysis should be reserved in very selected patient for whom timely percutaneous coronary intervention (PCI) is not feasible [52]. Thus, primary PCI is recommended as first-line therapy (IB level) according to ESC guidelines [52] (Fig. 3).

Another question of interest in AMI-CS was the strategy of revascularization in multivessel coronary artery disease (MVD). Indeed, up to 85% of AMI-CS patients present multivessel or left main coronary artery disease at the diagnostic time, MVD being associated with higher mortality compared to single-vessel disease [53]. Despite this, optimal management and timing of revascularization for non–infarct-related artery lesions in AMI-CS remains unclear. For decades ESC guidelines encouraged immediate multivessel percutaneous coronary intervention (MV-PCI) of all high-grade lesions, in addition to the culprit lesion with a class IIa C recommendation [52]. However, despite an appealing pathophysiological rationale, this approach was not supported by robust evidence [53, 54]. The debate seemed to be closed thanks to the recent and large randomized, multicenter Culprit Lesion Only PCI versus Multivessel PCI in Cardiogenic Shock (CULPRIT-SHOCK) [55]. Indeed, this trial showed a significant clinical benefit of a culprit-lesion-only strategy versus MV-PCI at early stage of CS with a reduction in the combined primary endpoint of 30-day mortality or severe renal failure requiring renal replacement therapy (45.9% vs. 55.4%; RR 0.83; 95% IC: 0.71–0.96; *p* = 0.01). Moreover, the culprit only strategy also provided an absolute 8.2% reduction in 30-day mortality (43.3% vs. 51.5%; RR 0.84; 95% IC 0.72–0.98, *p* = 0.03). These results were consistent across all predefined subgroups and were confirmed by a meta-analysis [56].

When patients at early stages of CS present non-revascularizable lesions by PCI and/or mechanical complications, there is a role for emergent coronary artery bypass grafting (CABG). In the IABP-Shock 2 study, 70% of patients with AMI-CS presented with MVD but only 4% underwent emergent CABG [57]. Few studies have addressed the issue of the benefit of surgical revascularization. Among them, few supported this strategy. Only observational data suggest that PCI and coronary artery bypass grafting could be similar in terms of mortality in AMI-CS [58] or even that CABG could confer better outcomes with the potential bias of patient selection [59]. Of note, improvements and better control of PCI techniques have led to decreased CABG indications.

To conclude, emergent revascularization in patients with coronary artery disease of culprit-lesion-only is the main treatment that improved CS prognosis. The question of the optimal timing of further revascularisation in patients with MVD who have undergone PCI of the infarct-related artery remains open.

#### Arrhythmia and conduction disorders management

Patients with CS have a high prevalence of arrhythmias and conduction disorders. The reported incidence of atrial fibrillation (AF) is as high as 20%, episodes of ventricular fibrillation occur in 9–19% of patients with CS [1]. In addition, 4.5–21% of patients will develop atrioventricular block [1] and 4.5% will require pacemaker implantation during hospitalization for CS [19]. No specific recommendations exist regarding supra-ventricular arrythmia management in CS. In patients with cardiogenic shock and arrhythmia, restoration of sinus rhythm, or slowing heart rate if restoration fails, can be useful to improve hemodynamic parameters. Thus, recent AHA Guidelines on acute AF management argue for immediate restoration of sinus rhythm in critically ill patients if AF is causing hemodynamic compromise. If the hemodynamic situation is stabilized, both rate and rhythm control are feasible [60]. As for the choice of pharmacological treatment, amiodarone has demonstrated its efficacy for pharmacological cardioversion in this population and seems to be the drug of choice in critically ill patients [61]. Of note, the paradigm of safe reduction within 48 h without anticoagulation has recently been challenged because recent data support an increased thromboembolic risk of AF reduction beyond 12 h of onset [62], this risk being correlated with the CHA_2_DS_2_-VASC score [63, 64].

The management of high-degree conduction disorders is not specific to patients with CS. For patients with sinus node dysfunction, second-degree or third-degree atrioventricular block associated with symptoms or hemodynamic compromise, atropine as a first line is reasonable to improve atrioventricular conduction, increase ventricular rate, and improve hemodynamic parameters (Class IIa recommandations). Furthermore, in patients with symptoms or hemodynamic compromise and who have low likelihood for coronary ischemia, beta-adrenergic agonists, such as isoproterenol, dopamine, dobutamine, or epinephrine, may be considered (Class IIb recommendation). When the high-grade conduction disorder is refractory to medical therapy, temporary transvenous pacing is reasonable (Class IIa recommendation) [65].

In patients with intraventricular conduction disorders, ventricular resynchronization can be considered in selected patients but only few reports describe its utilization in CS [66, 67]. In a single-center German study in 15 CS patients with left-bundle branch block, temporary resynchronization of the left ventricle using a lead placed in the coronary sinus optimized hemodynamic parameters and led to reduced blood-lactate plasmatic levels [68]. Of note, ESC Guidelines did not provide any recommendations on cardiac resynchronization therapy in a CS setting [34].

Finally, electrical storm is a life-threatening condition characterized by recurrent ventricular arrhythmia. A classical clinical definition of electrical storm is three or more sustained ventricular arrhythmia episodes (including appropriate ICD shocks) separated by at least 5 min over 24 h. The management of rhythmic storms should always include a cardiologist and must take several factors into account, namely the presence of underlying pro-arrhythmogenic cardiopathy, previously known left ventricular function, the presence of an implantable cardioverter-defibrillator and hemodynamic tolerance. In addition, the search for a myocardial ischemia substrate must be systematic [69]. In CS patients (i.e., with hemodynamically unstable ventricular arrhythmia), immediate electrical cardioversion is always preferred for patients and is appropriate for hemodynamically stable VT when the risk of sedation is low. IV Amiodarone is recommended as the first-line anti-arrhythmic therapy in electrical storm caused by structural heart disease in the absence of QT prolongation or Torsade de pointe, because of its greater efficacy for preventing recurrent VT and suppressing VT refractory to other anti-arrhythmic therapy. The recommended dose is a bolus og 300 mg (max 5 mg/kg) over 20 min then repeat 150 mg bolus over 10 min for recurrent ventricular arrhythmia, then Infusion 1 mg/min until free from ventricular arrhythmia ≥ 6 h (may continue for longer) and continue 0.5 mg/min until electrical storm resolves. When amiodarone is ineffective as monotherapy or for higher-risk presentations, lidocaine is often added as a second-line therapy during further amiodarone loading [initial bolus of 1–1.5 mg/kg (max 100–120 mg)]. Of note, lidocaine is more effective on an ischemic myocardium but can accumulate during decompensated heart failure or CS [70]. Otherwise, specific electrical storm etiologies and cardiomyopathies justify specific management, such as isoprenaline for Torsade de Pointe or Brugada syndrome and betablockers for Long QT syndrome.

Deep sedation and intubation is a very effective treatment of electrical storm [71] and is recommended in patients with an intractable electrical storm refractory to drug treatment (ClassIIa Recommandation, LOE, C). Moreover, implantable cardioverter-defibrillator interrogation is crucial to check frequency, effectiveness, and appropriateness of device therapies. Radiofrequency ablation as sympatholysis by stellar ganglion blockade and/or definite sympathectomy (by surgery or stereotaxic radiotherapy) should be considered in patients with recurrent episodes of ventricular arrythmia nonresponsive to medical treatment or coronary revascularization (ClassIIa Recommandation, LOE, C) [70, 72]. Finally, in eligible patients, heart transplantation may be considered with aMCS [73, 74].

#### Valvular disease management

The optimal management of severe valvular disease is challenging in CS patients because of the limited number of studies available. Several mechanisms may contribute to CS in the setting of decompensated valvular disease, and initial stabilization is recommended before evaluation for corrective surgery [75]. Cardiac surgery remains the gold-standard treatment in the management of symptomatic valvular disease but its invasiveness is associated with high mortality leading to consider alternative therapies such as percutaneous management. The role of percutaneous interventional management of valvular heart disease is increasing in CS patients as more and more data report its safety and good results in this severe population.

However, it is important to remember that in case of valvular deterioration due to endocarditis complicated by cardiogenic shock, surgery remains the cornerstone [76, 77].

In detail, in decompensated severe aortic stenosis, current guidelines are very elusive but keep a place for urgent valvuloplasty as a bridge to transcatheter aortic valve replacement (TAVR) or surgical aortic valve replacement (SAVR) [56, 57]. When aortic valvuloplasty is performed, the 1-year mortality remains dramatically high around 70% [78] while TAVR in CS patient is actually associated with 7.9–33% 1-month mortality [79–83]. A recent large observational real-world study by Goel et al. showed that adjusted 1-year mortality of TAVR was significantly higher at 29.7% in the CS group compared to 22.6% in the non-CS group (HR, 1.57; 95% CI 1.43–1.72; *p* < 0.001). However, patients who survived the first 30 days after TAVR had similar mortality rates to those who were not in CS [83].

Management of aortic insufficiency may include atrial pacing to increase heart rate and shorten diastole duration. SAVR is considered as the gold standard despite lack of data, but TAVR should be considered in selected patients [84].

In mitral regurgitation with CS, surgery is indicated for primary mitral disease but no evidence supports the benefit of surgery in secondary mitral regurgitation leaving a place for percutaneous approaches to the mitral valve [85–87]. In a large observational study, Simard and colleagues reported that transcatheter edge-to-edge mitral valve repair in patients with CS was feasible with excellent device success rate [in 3249 patients (85.6%)] with successful achievement of final mitral regurgitation grade ≤ 2 + in 88.2% of case [87]. Finally, severe mitral stenosis may be treated by percutaneous mitral valvuloplasty when surgery is not feasible [88, 89].

In summary, the indications for cardiac surgery in case of CS due to acute valvular disease should be personalized. In all cases, it seems important to discuss with experts and trained teams in a multidisciplinary approach (i.e., cardiologists, cardiac surgeons and intensivists). When medical treatment is the first-line choice, it also seems important to re-evaluate the interventional or surgical indication according to the evolution of CS and to reconsider the indication before the occurrence of refractory CS despite optimization of pharmacological treatment.

#### Inotropes/vasopressors

In patients with acute CS with hypotension, early vasopressors are recommended to increase perfusion pressure of the vital organs [27, 34, 35]. Most often in patients with acute-on chronic CS but also in the acute CS, inotropes are recommended to manage patients with hypotension and persistent low CO due to decreased LV systolic function, resulting in poor vital organ perfusion [27, 34, 35]. Most existing inotropes used in CS exert their physiological effects through the modulation of cardiomyocyte calcium flow inducing tachycardia. Thus, these drugs can increase myocardial oxygen consumption. These agents should be used with progressive titration at the lowest possible dose for the shortest duration. Relevant effects and mechanism of action of inotropes and vasopressors in CS setting are presented in Table 1.

**Table 1:** Inotropes and vasopressors use in cardiogenic shock

Agents	Mechanism of action	Main effects	Indications	Side effects
Norepinephrine	*α*_1+_ > *Β*_1 +_	Vasoconstriction	First-line vasopressor in cardiogenic shock	Excessive vasoconstriction, immunomodulation
Dobutamine	*Β*_1+_	Ionotropy, moderate vasodilatation	First-line inotropic agent in cardiogenic shock	Tachycardia, hypotension (excessive vasodilatation)
Milrinone	PD-3 inhibitor	Ionotropy	Second-line inotropic agent in CS	Tachyarrhythmias, hypotension, headache
Levosimendan	Myofilament calcium sensitizer	Ionotropy and inodilator	Second-line inotropic agent in cardiogenic shock (patients under betablockers)	Hypotension, atrial and ventricular tachyarrhythmias, headache

Moreover, the target mean blood pressure in CS is not well defined. Current recommendations are based on evidence in septic shock patients, suggesting that 65 mm Hg seems to be sufficient [27]. In CS, data are conflicting regarding the benefit of targeting a mean blood pressure higher than 65 mmHg [90–93].

During the last two decades, several studies have compared different regimens of vasopressors and inotropes, the most relevant of which are detailed below.

In the SOAP II trial, De Backer et al. evaluated the effects of two different regimens of first-line vasopressors in patients with shock and included a prespecified CS subgroup. Compared to norepinephrine, dopamine was associated with a higher rate of arrhythmias in the CS subgroup (280 patients) and in the overall population (1679 patients) and was associated with a higher risk of mortality in the CS subgroup [94]. The only randomized study (OPTIMA study) comparing, norepinephrine to epinephrine, reported the same hemodynamic efficacy with similar effects on arterial pressure and cardiac index, but epinephrine was associated with more arrhythmias, increased heart rates and lactic acidosis [95]. Furthermore, the OPTIMA study was stopped prematurely because of an increased incidence of refractory shock in CS patients receiving epinephrine [10 of 27 (37%) vs. 2 of 30 (7%); *p* = 0.008]. Finally, a meta-analysis including individual data from 2583 cardiogenic shock patients, among whom 37% (IQR 17–76%) received epinephrine, reported a three-fold increase in the risk of death with epinephrine (OR = 3.3 [2.8–3.9]) compared to other drugs [96]. In addition, in cardiac arrest patients with post-resuscitation shock, all-cause hospital mortality was found to be significantly higher when epinephrine was used (OR 2.6; 95% CI 1.4–4.7; *p* = 0.002) compared to norepinephrine. Cardiovascular hospital mortality was also higher with epinephrine (aOR = 5.5; 95% CI 3.0–10.3; *p* < 0.001) [97]. Given these data, norepinephrine is currently recommended as first-line vasopressor in CS [27, 34, 35].

Among intravenous inotropes, dobutamine, is recommended to improve myocardial contractility and cardiac output [IIb class recommendations] [34, 35]. In a pilot study on thirty patients, dobutamine resulted in less arrhythmia, less myocardial oxygen consumption, and a lower increase in lactate concentration compared with epinephrine [98]. Other inotropes that do not use the beta adrenergic receptor stimulation way (phosphodiesterase-3 inhibitors and levosimendan), have been assessed in CS patients because of their ability to improve myocardial contractility and their vasodilatory effect on pulmonary artery without increasing myocardial oxygen consumption. Milrinone (half life time of 2 h) decreases the rate of intracellular cyclic adenosine mono-phosphate breakdown, which increases intracellular calcium, myocardial contractility, and cardiomyocyte relaxation. A randomized study (DOREMI trial) on 192 participants (96 in each group), had recently compared milrinone with dobutamine in the management of CS. The primary composite outcome (in-hospital death, resuscitated cardiac arrest, receipt of cardiac transplant or mechanical circulatory support, nonfatal myocardial infarction, stroke or initiation of renal replacement therapy) did not differ between the milrinone [47 of 96 (49%)] and dobutamine group [52 of 96 (54%); relative risk, 0.90; 95% CI (0.69–1.19); *p* = 0.47]. Furthermore, no significant differences were found in heart rate, mean arterial pressure, serum lactate level, hourly urine output or serum creatinine level between the treatment groups [99].

Levosimendan seemed promising because it improves myocardial contractility by increasing myofilament calcium sensitivity, without raising intracellular calcium and AMP concentrations. However few data support its use in CS and they suffer from important methodologic bias. In the SURVIVE Study, 1227 patients suffering from acute-on-chronic CS were randomized to rceive either intravenous levosimendan (*n* = 664) or intravenous dobutamine (*n* = 663). No difference was found regarding all-cause mortality at 180 days. There were higher incidences of atrial fibrillation, hypokalemia, and headache in the levosimendan group [100]. A systematic Cochrane database review found that levosimendan compared to dobutamine did not reduce short-term mortality (RR = 0.60, 95% CI 0.36–1.03; participants = 1701; low-quality evidence) and long-term mortality (RR = 0.84, 95% CI 0.63–1.13; participants = 1591; low-quality evidence); and did not reduce long-term mortality versus placebo (long-term mortality: RR 0.55, 95% CI 0.16–1.90; participants = 55; very low-quality evidence, no data on short-term mortality) [101]. Other studies reported a possible increased risk of complications including adverse cardiovascular events and excessive peripheral vasodilatation with hypotension [102]. A meta-analysis assessing data from 5,480 patients in 45 randomized clinical trials reported a benefit of levosimendan on mortality (risk ratio = 0.80 [0.72; 0.89], *p* for effect < 0.001). However, in this meta-analysis, trials on patients with acute cardiac events were combined with trials on patients undergoing elective cardiac surgery [103]. Of note, Levosimendan may be useful in CS patients on chronic beta-blocker treatment. In a subgroup analysis from the SURVIVE study in patients who used beta-blockers (*n* = 669), mortality was significantly lower for levosimendan than dobutamine at day 5 (1.5 vs. 5.1% deaths; HR = 0.29; (0.11–0.78), *p* = 0.01) [104].

#### Mechanical ventilation

The incidence of acute respiratory failure (ARF) in CS patients ranges between 50 and 88% with more than 80% of patients with CS requiring respiratory support [19, 20]. Cardiogenic pulmonary edema is backward failure due to LV systolic and diastolic dysfunction [105]. This excess of interstitial and alveoli fluids impairs ventilation; and increases work of breathing, which ultimately results in both hypoxemia and hypercapnia.

In addition, intrapleural pressure (Ppleural) seems to be a major determinant of respiratory impairment. During respiratory failure, active respiratory muscles contraction induces a negative Ppleural, increasing venous return and RV/LV preload [106]. Other factors contribute to a further impairment in gas exchange. Indeed the concurrent inflammatory response conduces to the development of alveolar edema by changing vascular membrane permeability; while acute kidney injury favors fluid retention [105]. The compensatory increase in respiratory drive in response to hypoxia, hypercapnia and/or metabolic acidosis, can further redirect cardiac output toward respiratory muscles, perpetuating myocardial ischemia and tissue hypoperfusion in CS patients [107].

These pathophysiological concepts support the use of ventilatory support with positive pressure ventilation (PPV) for management of CS patients with respiratory distress [106]. The first aim of the ventilatory support with PPV is to improve gas exchanges (i.e., oxygenation and decarboxylation) as it reduces alveolo-interstitial edema and enhances alveolar recruitment [108, 109]. It leads to a better systemic and myocardial oxygenation and reversion of hypoxic pulmonary vasoconstriction. PPV decreases myocardial oxygen demand by decreasing the myocardial wall tension (Laplace law). In addition, PPV also decreases work-of-breathing and reduces the oxygen consumption of the diaphragm. Finally, PPV leads to favorable hemodynamic effects by reducing RV/LV preload and LV afterload and therefore improves CO [107, 110].

Modalities of different respiratory supports use in cardiogenic shock are displayed in Table 2. Data regarding respiratory management of acute cardiogenic pulmonary oedema came from studies that were not dedicated to cardiogenic shock. In a large multicentric randomized study comparing oxygen therapy versus noninvasive ventilation in acute cardiogenic pulmonary edema, PPV induced a faster improvement of respiratory distress and acidosis. There was no significant difference in mortality or intubation within 7 days. However, these results suffer from limitations regarding the low intubation and mortality rate [109]. Recent studies also suggested that PPV with noninvasive ventilation reduced the risk of invasive mechanical ventilation (IMV) [111–113].

**Table 2:** Modalities, settings, advantages, limits and indications of the different respiratory supports used in cardiogenic shock

Modalities	Settings	Advantages	Limits	Indications
O2 mask	Oxygen flow: adjust with objective of SaO_2_ 92–98%	Avoid hypoxia Simple and accessible device	No PEEP effect Limited oxygen flow at 15 L/min	First-line support until NIV or IMV in case of respiratory failure Acute cardiogenic pulmonary edema: compared to oxygenotherapy, PEEP induced a faster improvement of respiratory distress and acidosis without significant difference in mortality or intubation within 7 days [109] No data regarding CS specifically
Non invasive ventilation (NIV) Continuous positive airway pressure (CPAP) Bilevel positive air pressure (BIPAP)	Positive Pressure ventilation (PPV) with positive end expiratory pressure (PEEP): minimal starting setting at 5–10 cmH_2_O, adjust according to SaO_2_ In case of BIPAP, adjust inspiratory positive airway pressure according to respiratory rate FiO2: adjust with objective of SaO_2_ 92–98% [106] Need correct selection of interfaces, favorable patient/ventilator synchrony, comfort and active participation of the patient	1. Respiratory effects: Reduced alveolo-interstitial edema Induced alveolar recruitment Improve oxygenation: reversion of hypoxic pulmonary vasoconstriction, better systemic and myocardial oxygenation Improve decarboxylation Decreasing work-of-breathing and reduce the oxygen consumption of the diaphragm 2. Hemodynamic effects Reduce RV/LV preload and LV afterload Improve cardiac output Could reduce the risk of invasive mechanical ventilation [111–113] Simple and accessible device	Absolute contraindications: Severely impaired consciousness Refractory vomiting Facial trauma Hemodynamic instability Relative contraindications: Inability to cough Uncooperative patient NIV compared to IMV in a non-randomized study: no difference of mortality [115]	First line: respiratory distress in heart failure patients, without absolute contraindications to NIV [5, 34, 35, 106] Second line: should be considered in CS only after: Hemodynamic stabilization An assessment of the risk–benefit balance Without absolute contraindications to NIV With a close monitoring of efficacity Device: BIPAP increase Vt and could be preferable compared to CPAP in case of hypercapnia, chronic lung disease or severe RF
Invasive mechanical ventilation (IMV)	FiO2: adjust with objective of SaO_2_ 92–98% [106] PEEP: set at 5 cmH_2_O, gradually increased according to respiratory (overdistension, barotrauma) and hemodynamic (decrease RV preload and increase RV afterload) risks of excessive PEEP Respiratory rate: adjust to limit hypercapnia acidosis (worsening of RV dysfunction risk) Vt: should not exceed 6–8 mL/kg of ideal body weight No data and recommendations regarding superiority of any specific mode (volume or pressure control) [5, 34, 35, 106]	1. Respiratory effects: Reduced alveolo-interstitial edema Induced alveolar recruitment Improve oxygenation: reversion of hypoxic pulmonary vasoconstriction, better systemic and myocardial oxygenation Improve decarboxylation Decreasing work-of-breathing and reduce the oxygen consumption of the diaphragm 2. Hemodynamic effects: Reduce RV/LV preload and LV afterload Improve cardiac output Sedation use: could reduce oxygen consumption and improve cardiac output	Risks associated with anaesthesia induction and intubation: hypotension, hypoxia, transient low cardiac output due to intrathoracic pressures change, cardiac arrest Specific mode: pressure supported ventilation (PSV) could increase myocardial oxygen consumption if spontaneous breath is no fully supported [122]	First line: [5, 34, 35, 106] Refractory shock with severe hypotension Need for effective airway protection due to coma/impairment of consciousness Refractory vomiting Facial trauma Second line after NIV failure [5, 34, 35, 106]
High flow nasal canula (HFNC)	FiO2: adjust with objective of SaO_2_ 92–98%	High oxygen flow (max 60L/min, FIO2 1) Improvement of oxygenation Simple and accessible device	Inconstant PEEP “effect”, between 5 and 7 cmH_2_O	Scarce data Remains actually unclear [119, 120]

BIPAP, bilevel positive air pressure; CS, cardiogenic shock; CPAP, Continuous positive airway pressure; PPV, Positive Pressure ventilation; NIV, Noninvasive ventilation; PEEP, positive end expiratory pressure; RV, right ventricular; LV, left ventricular; Vt, tidal volume; PSV, pressure supported ventilation

NIV represents a cornerstone in the management of cardiogenic pulmonary edema but its place in CS management is more questionable, mainly due to the hemodynamic instability or consciousness impairment. Importantly, there is no randomized study that compared NIV with IMV in this population. Recent European Guidelines regarding the management of respiratory distress in heart failure patients suggested to use NIV before intubation but do not address specifically the issue of CS patients [114]. The expected benefit of IMV is the reduction of oxygen consumption by sedation, allowing the optimization of CO [105]. One observational study including 219 CS patients compared NIV with IMV and highlighted no difference of mortality after propensity score adjustment [115]. These data suggest to consider NIV with caution after assessment of the risk–benefit balance. Refractory shock with severe hypotension and the need for effective airway protection due to coma are absolute contraindications to NIV. In addition, mild hypotension, inability to expectorate copious secretions, uncooperative patient and isolated RV failure should be considered as relative contraindications [108]. In consequence, the use of NIV should be considered only after hemodynamic stabilization with a close monitoring of its efficacy, that is usually observed within 1–2 h from its initiation [105, 114]. Overall, success of NIV is conditioned by correct selection of interfaces, favorable patient/ventilator synchrony, comfort and active participation of the patient [116].

In practice, two main modalities of NIV are used in ICU, i.e., continuous positive airway pressure (CPAP) and bilevel positive air pressure (BIPAP) with positive end expiratory pressure (PEEP). Minimal starting setting of PPV should be 5–10 cmH_2_O, adjusted according to oxygen saturation (SaO_2_). Actually, CPAP and BiPAP can be used indifferently, depending on the practice of the centers, in the absence of demonstrated difference in mortality and intubation rates [109, 117]. Although CPAP is a simpler technique, BIPAP could increase tidal volume and seems to be preferable in patients with hypercapnia, chronic lung disease or severe respiratory failure. Finally, high flow nasal canula shows improvement of oxygenation, providing an inconstant PEEP “effect” between 5 and 7 cmH_2_O [118]. Considering the paucity of data in the respiratory management of CS, the place of this device remains actually unclear [119, 120].

When IMV is needed, patients with cardiogenic shock should benefit from early intubation. A recent study highlighted that each 1-h delay in IMV initiation was associated with higher 30-day mortality (OR, 1.03; 95% CI 1.00–1.06; *p* = 0.03) [121]. However, particular attention in early intubation should be paid to cases of severe right ventricular dysfunction or tamponade. In these cases, PPV has to be used only when necessary, optimizing preload and MAP (> 60 mmHg) with vasopressors is needed and consider awake intubation if tamponade/constriction.

In CS patients, no data support the superiority of any specific mode (volume or pressure control, pressure supported ventilation [106]. Importantly, spontaneous breathing modes as PSV could increase myocardial oxygen consumption if spontaneous breathing is not fully supported, making this ventilation mode less suitable to the CS acute phase [122]. PEEP level is set at 5 cmH_2_O and gradually increased taking into account the respiratory (overdistension, barotrauma) and hemodynamic (decrease RV preload and increase RV afterload) risks of excessive PEEP [106]. FiO2 may be titrated to a goal of SaO_2_ between 92 and 98% [106]. The rationale is to avoid either hypoxemia and hyperoxemia. Of note, the ideal oxygenation targets in CS patients remain undefined in current guidelines [5, 34, 35], but emerging evidence highlights the necessary to avoid hyperoxemia, as SaO_2_ > 98%, that was found to be deleterious [123–125]. Respiratory rate is mainly adjusted to limit acidosis, as hypercapnia could worsen RV dysfunction. Finally, tidal volume (Vt) > 9 mL/kg seems to be associated with higher mortality (OR 9.0, 95% CI 1.3–62.0, *p* = 0.03), suggesting that Vt setting should not exceed 6–8 mL/kg of ideal body weight [126].

#### Renal replacement therapy

Acute kidney injury (AKI) occurs in up to one half of patients with CS, and 20% of these patients will require renal replacement therapy (RRT) [127]. AKI is a strong predictor of poor outcomes regardless of the etiology of CS [127, 128]. Indeed, patients with an indication for RRT have a two-fold increase in the risk of in-hospital mortality [127, 129, 130] along with a higher risk of long term dialysis when compared with those with no need for RRT [129]. In a recently published study, age, lactate, hemoglobin, pre-admission loop diuretics use and baseline eGFR were identified as the most important clinical predictors of poor outcomes in patients with CS requiring RRT [131].

In patients with acute decompensated heart failure with worsened renal function but without shock, RRT with ultrafiltration was associated with improved hemodynamic parameters but also with a higher incidence of adverse events and worsening of renal function and transition to dialysis [132, 133]. Recent AKIKI studies on critically ill patients with shock and AKI demonstrate no difference in terms of mortality of a delayed strategy versus an early RRT initiation strategy [134, 135]. These results highlighted that RRT may be deferred until one of these criteria is met: severe hyperkalemia, metabolic acidosis, pulmonary edema, blood urea nitrogen level higher than 112 mg per deciliter, or oliguria for more than 72 h.

To date, no clear recommendation exists for the ideal timing for RRT initiation in patients with CS. Available studies in this regard are scarce and limited to the post-cardiac surgery setting. In a retrospective analysis of 142 patients managed with post cardiotomy CS complicated with AKI, the early initiation of high-volume continuous veno-venous hemofiltration resulted in improved outcomes with lower in-hospital and 30-day mortality rate when compared to patients undergoing low volume hemofiltration [136]. Importantly, as patients with CS do not tolerate acute fluid shifts as observed in patients with intermittent hemodialysis, continuous RRT is the most commonly used strategy [5].

#### Acute mechanical circulatory support

In CS, short-term acute mechanical circulatory support (aMCS) should be considered when urgent hemodynamic stabilization is needed despite ongoing medical therapies to allow for heart recovery and/or end-organ protection (as a bridge to recovery), or as a bridge to transplantation or long-term aMCS (as a bridge to a bridge or bridge to decision strategy) in appropriately selected patients, as per the 2022 AHA (Class IIa, Level of Evidence B-NR) and the 2021 ESC (IIa, C) recommendations [34, 35, 137]. Emerging data suggest that prompt implantation of aMCS devices in well-selected patients in whom decision-making is based on early invasive hemodynamic assessment and standardized treatment algorithms may improve outcomes [36, 138, 139]. However, evidence for sustained survival benefits with short-term aMCS is limited [121] and to date studies, such as IABP-SHOCK II, comparing intra-aortic balloon pump (IABP) with other MCS devices like Impella, show no significant differences in mortality [42, 122–125]. Importantly, there is an absence of proof to date to prefer one to another aMCS and device selection depends on various factors, including CS phenotype and local expertise [9]. Practices on aMCS type use vary from country, particularly in France where the veno-arterial extracorporeal membrane oxygenation (VA-ECMO) is predominantly used, whereas the Impella device is becoming increasingly popular in the US and Germany, for example. In a recent large nationwide database in US, 20% of AMICS patients received an aMCS of which 66, 22.7 and 10.5% were Impella, VA-ECMO or combination of both devices, respectively [144]. Recent data emphasize the importance of early aMCS escalation, especially in MI-related CS. Registry findings suggest improved survival with early MCS implantation before percutaneous coronary intervention (PCI) [5, 9, 34, 35].

VA-ECMO use has increased, with varying survival rates based on etiology [145–149]. Randomized studies like EURO-SHOCK [150], ECMO-CS and ECLS-SHOCK trial showed mixed results, and ongoing trial ANCHOR (NCT04184635) aims to provide further insights. In detail, results of the first randomized study were recently published (ECMO-CS): among 122 patients, there was no improvement, in patients with CS supported with early VA-ECMO, in the primary composite outcome (death from any cause, resuscitated circulatory arrest, and implementation of another mechanical circulatory support device at 30 days): 37 (63.8%) patients in the immediate VA-ECMO and 42 (71.2%) in the no early VA-ECMO groups, respectively (hazard ratio, 0.72 [95% CI 0.46–1.12]; *p* = 0.21. All-cause mortality was 50.0% in immediate ECMO group versus 47.5%; risk difference, 2.5 [95% CI − 15.6 to 20.7]; while the cross-over rate was 39% in no-early ECMO group [151]. Finally, in a larger randomized study (ECLS-SHOCK trial) conducted by Thiele and colleagues on 417 patients with an acute myocardial infarction complicated by cardiogenic shock, there was no difference on death from any cause at 30 days (the primary end point) that occurred in 47.8% of the patients in the ECLS group and in 49.0% of those in the control group (relative risk, 0.98; 95% confidence interval, 0.80–1.19; *p* = 0.81). Moreover, the use of VA ECMO was associated with increased side effects including bleedings [152]. The meta-analysis of four randomized clinical trials comparing early routine use of VA-ECMO versus optimal medical therapy alone in patients presenting with infarct-related cardiogenic shock trials did not report any significant reduction of 30-day death rate with the early use of VA-ECMO (OR 0.93; 95% CI 0.66–1.29) [153]. These data rule out the use of VA-ECMO in most patients with CS related to myocardial infarction. Further studies are needed to determine whether there is a sub-group of these patients in whom the benefits outweigh the harms, namely bleedings and vascular complications [154].

Finally, VA ECMO resulting in increased LV afterload, this may lead to inadequate unloading of the LV. Combining veno-arterial ECMO with IABP, Impella support, or atrial septostomy could be considered to achieve LV unloading [155]. Accordingly, some observational studies showed that these strategies of adding an IABP or Impella to peripheral VA-ECMO was associated to an increase in survival [156, 157].

## Conclusions

The management of CS has seen significant advances in recent decades, but the mortality remains dramatically high. As for other types of shock, rapid recognition with multimodal evaluation by ECG, biological and echocardiographic tests allows early and appropriate management and may improve survival. Recent classifications allowing better stratification of mortality risk may be useful to guide the management. The cornerstone of CS patient management is the treatment of the cause of the cardiac insult including coronary revascularization by PCI of the culprit lesion in case of AMI. Symptomatic management aims to restore perfusion with noradrenaline and increase cardiac output with inotropic therapies. The management of other organs failure is based on optimization of mechanical ventilation and appropriate initiation of RRT. Mechanical assist devices may have an important role in CS patients but, pending the results of further clinical trials, the type and implantation timing of such techniques remain to be defined according to the expertise of the Heart Team.

## Data Availability

Not applicable.

## Abbreviations

AMI: Acute myocardial infarction
AMI-CS: Cardiogenic shock secondary to acute myocardial infarction
AKI: Acute kidney injury
BIPAP: Bilevel positive air pressure
CABG: Coronary artery bypass grafting
CO: Cardiac output
CPAP: Continuous positive airway pressure
CS: Cardiogenic shock
IABP: Intra-aortic balloon pump
ICU: Intensive care unit
IMV: Invasive mechanical ventilation
LV: Left ventricular
aMCS: Acute mechanical cardiac support
NIV: Non-invasive ventilation
PCI: Percutaneous coronary intervention
RRT: Renal replacement therapy
RV: Right ventricular
SAVR: Surgical aortic valve replacement
TAVR: Transcatheter aortic valve replacement
TTE: Transthoracic echocardiography
VA-ECMO: Veno-arterial extra-corporeal membrane oxygenation
